# Database of Italian present-day stress indicators, IPSI 1.4

**DOI:** 10.1038/s41597-020-00640-w

**Published:** 2020-09-08

**Authors:** Maria Teresa Mariucci, Paola Montone

**Affiliations:** Istituto Nazionale di Geofisica e Vulcanologia, Rome, Italy

**Keywords:** Geodynamics, Geophysics, Tectonics

## Abstract

The Italian Present-day Stress Indicators (IPSI) database is a freely available Italian georeferenced repository of information regarding the crustal stress field. It consists of horizontal stress orientations that have been analysed, compiled in a standardised format and quality-ranked for reliability and comparability on a global scale. The database contains a collection of information regarding contemporary stress within the shallow crust from the following main stress-indicator categories: borehole breakouts; earthquake focal mechanisms; seismic sequences and active fault-slip data. The present database (IPSI 1.4) released in January 2020 is accessible through a web interface which facilitates findability, accessibility, interoperability and reusability of the dataset. Moreover, it contains 928 records updated up until December 2019 with an increase of 10% with respect to the first one, and improved metadata information. The uniform spread of stress data over a given territory is relevant for earth crustal modelling or as starting point in many applied studies. It is therefore necessary to continue collecting new data and update present-day stress maps to obtain more reliable evaluations.

## Abstract (summary)

**Table:** 

Measurement(s)	current earth crustal stress orientation
Technology Type(s)	digital curation
Factor Type(s)	type of stress indicator • region of data collection • time of data collection
Sample Characteristic - Environment	planetary crust
Sample Characteristic - Location	Italy

Machine-accessible metadata file describing the reported data: 10.6084/m9.figshare.12820508

## Background & Summary

**Fig. 1:**
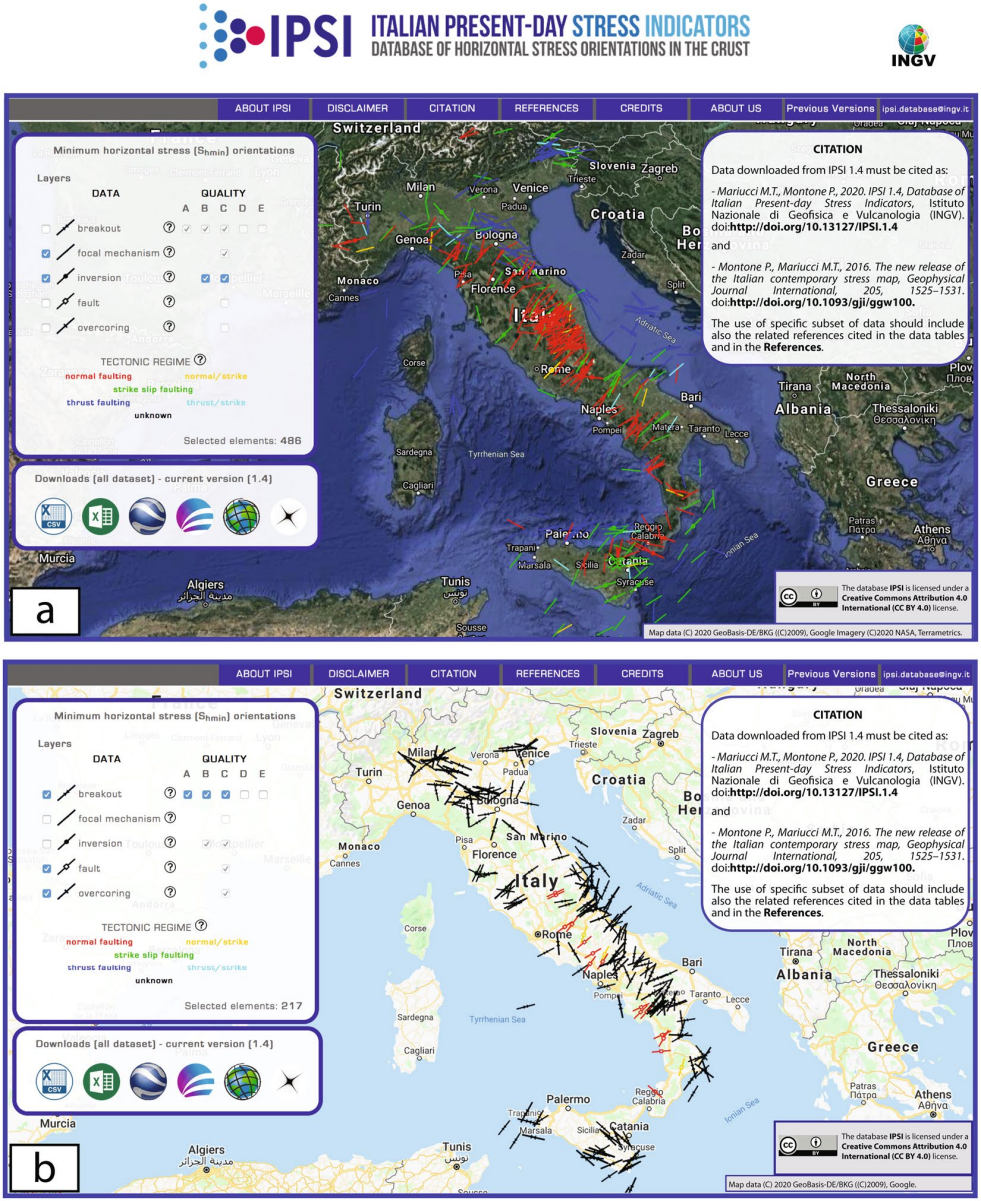
The Italian Present-day Stress Indicators database (IPSI 1.4)^2^. Stress data can be plotted by selecting the indicator type and quality on the left. (**a**) Example of focal mechanisms and inversions in the satellite map viewer. (**b**) Example with breakout, fault and overcoring data in the road map viewer. All datasets can be downloaded by choosing between six different file formats in the lower left box.

The Italian Present-day Stress Indicators (IPSI) database (http://ipsi.rm.ingv.it/) is the first geo-referenced repository of crustal contemporary stress field orientations in Italy relative to borehole breakouts from deep wells, earthquake focal mechanisms, faults and overcoring data (Fig. 1). Since 2017^1^, the database has been maintained at the Istituto Nazionale di Geofisica e Vulcanologia (INGV) and now contains 928 records updated up until December 2019^2^.

Contemporary stress data are important for several applications and an updated stress map can be successfully used by several users working on a specific topic. Current stress data are invaluable in seismic hazard assessment, for the identification and investigation of local stress perturbations, improvement of knowledge regarding the tectonic setting of the region and to constrain geophysical and geodynamic models. On a smaller scale, stress data support rock mechanics experiments for understanding processes linked to faulting and earthquakes. This is particularly significant in Italy, which is situated in a complex tectonic setting. Although the dataset is substantial on a large scale, the stress data distribution in Italy highlights some areas that show changes in stress regime over small distances and/or with depth^3,4^. Where information is lacking, each stress pattern prediction could accordingly differ considerably from reality and further evaluation may only be weakly supported by the data. For this reason, we continuously analyse and collect stress indicators and make them available online.

Italian stress data are regularly delivered to the World Stress Map Project (WSM), a global compilation of present-day crustal stress data that have been maintained at the Helmholtz Centre Potsdam GFZ since 2009^5^ and began in 1986 within the International Lithosphere Program^6^. IPSI stress data are analysed and quality-ranked according to the WSM standardised methods to make the two dataset fully comparable.

The analysis of stress indicators in Italy began in early 1990s along the coastal Tyrrhenian region of central Italy. In particular, 15 geothermal wells were analysed to detect borehole breakouts and compare them with stress directions inferred from the inversion of microearthquake focal mechanisms^7^. Despite the presence of thousands of wells drilled in Italy, breakouts were almost completely absent in the WSM database except for a few data analysed in Sicily^8–10^. Following these first encouraging results, we began to analyse borehole breakouts in deep wells in southern Italy^11,12^ and produced the first crustal stress regime map with data from about 200 wells and from earthquake focal mechanisms^13^. By the end of 1990s, some specific papers were published on the recent tectonic evolution and present stress field in northern Italy^3,14^, followed by a new Italian stress map with about 350 stress data points^15^.

Before the release of a new map of Italy and surrounding regions containing about 540 entries^16^, some studies were developed that analysed southern Italy data^4,17^. The growing quantity of stress information allowed the Italian tectonic regime to be assessed^18^, a comparison between active stress and local tectonic structures^19,20^ and the first modelling of the present-day stress field in southern Italy, which also included GPS data^21^.

Following the Mw 6.3 2009 L’Aquila earthquake (central Italy)^22^, two deep wells were analysed to recognise stress orientations and highlight the relationship with the seismogenic fault^23^. Stress data have also been used to constrain the geometry of a seismogenic fault in southern Italy^24^.

In 2012, a significant update of the Italian present-day stress map, with the inclusion of more than 700 stress indicators, allowed for highlighting small-scale changes in stress orientation^25^. With respect to the previous compilation, the most recent version^26^ contained more than 850 entries to define the stress orientation and tectonic regime in areas that were previously poorly covered, especially those affected by the 2012 Emilia seismic sequence^27^. Recent studies have addressed earthquake focal mechanism forecasting, and seismogenic sources have been realised using the latest stress dataset^28,29^.

In this work, we present the last version of the database whose web interface facilitates the findability, accessibility, interoperability and reusability of the database. This new version contains about 10% of more data and an improved collection of metadata description.

## Methods

**Fig. 2:**
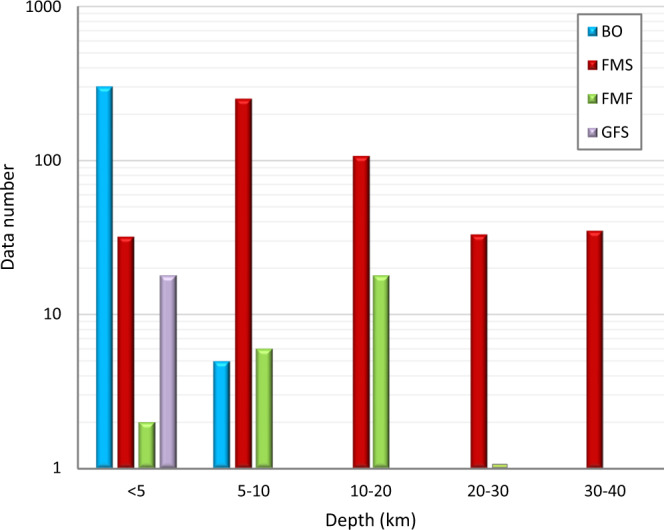
Depth distribution of stress data type. BO, borehole breakouts; FMS, earthquake focal mechanisms; FMF, formal inversions; GFS faults. Data number in logarithmic scale.

The IPSI 1.4 database contains information collected up until December 2019 concerning horizontal stress orientations inferred from different stress indicators^2^. Data are relative to borehole breakouts from deep wells, crustal earthquake focal mechanisms and fault data. All data refer to a depth interval down to 40 km, that corresponds to an average maximum crustal thickness in Italy. The depth ranges of the different stress indicators are almost complementary, as faults yield stress information about the surface, breakouts in the first kilometers and earthquakes mostly in the deepest ranges (Fig. 2), providing a complete picture of crustal present-day stress field.

In particular, IPSI 1.4 includes more than 10% data with respect to the first version of the database^1,26^, relatively to focal mechanisms, formal inversions and faults mainly located in central Italy. In addition to the bibliographic references associated with the single data, it contains all the information on the available wells (stratigraphy and geophysical logs), and since 2018, we have no longer considered Centroid Moment Tensor earthquake solutions but those obtained from high-quality data^30^.

**Fig. 3:**
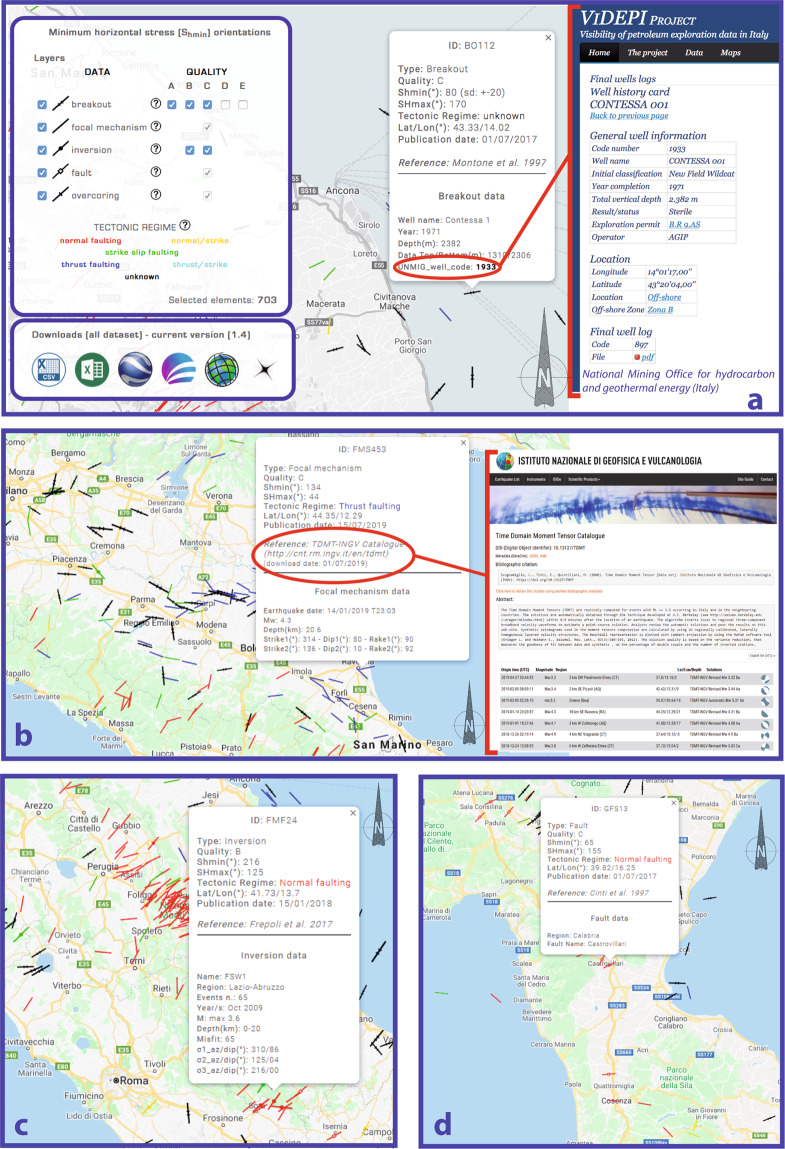
Examples of the IPSI website^2^. Pop-up windows provide summary information about the selected element. (**a**) Borehole breakout: red contour line evidences the link to the well database of the Italian Ministry of Economic Development (https://www.videpi.com). (**b**) Earthquake focal mechanism: red contour shows the link to the focal mechanism catalogue with data used to infer the stress orientation. (**c**) Formal inversion. (**d**) Fault. See diagram (**a**) for symbol legend.

IPSI was mainly conceived for users interested in studying the stress field of the Italian crust in a more accurate and complete way. Most of the data are also part of the WSM (http://www.world-stress-map.org/), as evidenced by many links that redirect to this database, and all these data are regularly delivered to the WSM project. The IPSI website (http://ipsi.rm.ingv.it/) provides access to data in a standard map viewer where data can be selected for plotting (category and/or quality) and downloaded in common file formats (Fig. 1). The legend on the left shows the basic information of the different data, the tectonic regime assignment and quality ranking method with pop-up windows and linked files. The main information of each element (type, quality, orientation) can be viewed by hovering over the related symbol on the map and more details appear by clicking the selected element (Fig. 3).

In this section, we describe the methods used to analyse the different indicators for defining stress orientations. According to the WSM ranking scheme^31^, a quality category (*A* to *E*, from best to worst) is assigned to each stress data orientation record. Quality *A–C* indicates that the stress orientation is within ± 15°, ± 20° and ± 25°, respectively, *D*-quality means that stress orientation is questionable (within ± 40°) and *E*-quality denotes unreliable or insufficient information.

The stress regime expresses the relative magnitude of the three principal stress axes (S1, S2 and S3). Stress magnitudes are defined using standard geologic/geophysical notation with positive compressive stress: S1 as the maximum, S2 as the intermediate and S3 as the minimum principal stress axis. Assuming that one of the principal stresses is vertical (Sv), SHmax and Shmin are the maximum and minimum principal components of the stress tensor on the horizontal plane, respectively.

**Fig. 4:**
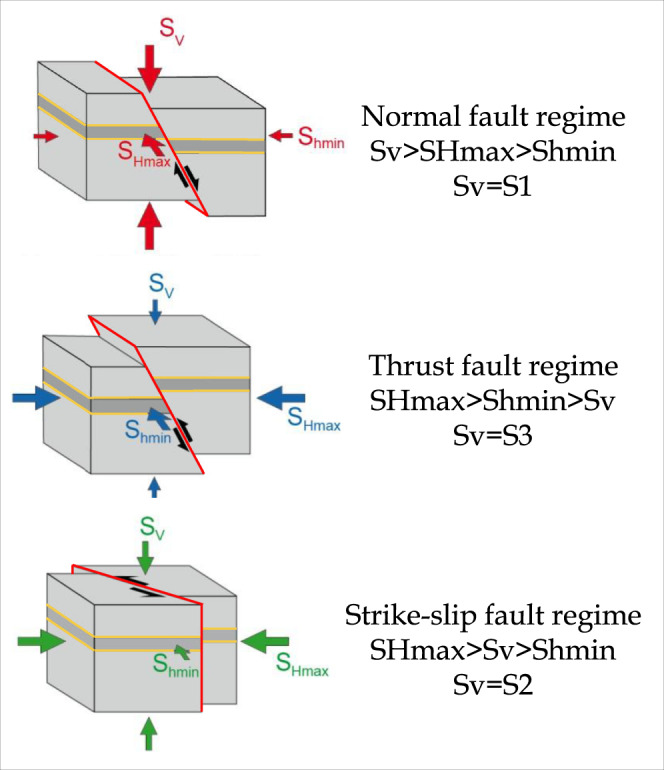
Main faulting and tectonic regime categories according to ref. ^32^.

As related to fault kinematics, the main categories of a tectonic regime are thrust, normal and strike-slip faults^32^. Only when faults are optimally oriented with respect to the stress field is the stress regime coincident with the tectonic regime^33–35^. In normal faults, S1 is vertical, Shmin corresponds to S3 and SHmax to S2. In thrust faults, S3 is vertical, Shmin corresponds to S2 and SHmax to S1. In strike-slip faults, S2 is vertical, Shmin corresponds to S3 and SHmax to S1 (Fig. 4).

On the IPSI website, we report results in terms of the minimum horizontal stress orientation on the map (corresponding to either S2 in thrust regime or S3 in a normal or strike-slip regime), and both Shmin and SHmax in the data records. We classify the data, except for borehole breakout data, into five tectonic regime categories: normal faults (NF); thrust faults (TF); strike-slip faults (SS); normal faults with a strike-slip component (transtension, NS) and thrust faults with strike-slip component (transpression, TS) according to the WSM categorisation^6^.

The database also includes a single overcoring datum that has been taken from WSM^36^. Usually overcoring data are referred to a very shallow depth and then comparable to the fault data. For a more complete description of overcoring technique please refer also to ref. ^37^.

### Borehole breakout data

Borehole breakouts are stress-induced “enlargements” of a wellbore cross-section that occur discontinuously when a well is drilled in rocks within an anisotropic stress field^38,39^. The “enlargements” develop on opposite sides of the borehole wall along the Shmin direction. Following the main criteria reported in refs. ^40–42^, we determine breakouts using records from a four-arm caliper tool in deep wells (approximately 0.450–7 km depth). We take into account wells with deviation not more than 15° and not less than 0.5° from the vertical, usually the deviation is much less than 10°. We apply the circular statistics of ref. ^43^ to compute the mean breakout orientation and standard deviation (95% confidence) for each borehole weighed by the breakout zone length. Up to now we have not been able to use image logs that would reduce the uncertainty in breakout orientations, as shown by recent studies (e.g. ref. ^44^).The WSM quality ranking system^31^ (*A* to *E*) used to classify the breakout orientation of each well accounts for the number of breakout zones, total length of the breakouts and standard deviation of the orientation (Table 1). We assign *E*-quality to well data without reliable breakouts (standard deviation > 40°) or evidencing no breakouts along the borehole, therefore stress orientations and related information are not provided in the database. We always analyse in detail the possible reasons of such results, especially in order to verify if some stress orientations with large standard deviation could be related to stress rotations nearby major fault zones.

**Table 1:** Grid for quality ranking of borehole breakouts (BO) inferred from caliper logs, according to WSM criteria^31^.

BO zones/BO length along a well	Standard deviation (sd) of average BO orientation in a well				
	sd ≤ 12°	12° < sd ≤ 20°	20° < sd ≤ 25°	25° < sd ≤ 40°	sd > 40°
10/>300 m	A	B	C	D	E
6/100–300 m	B	B	C	D	E
4/30–100 m	C	C	C	D	E
<4/<30 m	D	D	D	D	E

Borehole breakout orientations alone do not allow evaluation of tectonic regime. They can be combined with other borehole data, for instance along with rock strength or together with leak off tests to estimate or constrain stress magnitude and infer faulting regime (e.g. refs. ^45–47^).

Once selecting an element on the IPSI website, a pop-up window provides the related identification code and summary information from the data record. The upper part includes the type, quality, stress orientation, tectonic regime, geographic coordinates and date of the first online publication and its reference location. The lower part includes additional information such as the well name where the breakout was inferred, drilling year, vertical well depth, top and bottom of the breakout data and code of the well in the National Mining Office database for hydrocarbon and geothermal energy (UNMIG) of the Italian Ministry of Economic Development. The latter includes a link to the Videpi archive (https://www.videpi.com) where well stratigraphic log can be viewed and downloaded (Fig. 3a). Usually, associated to the stratigraphy there are some geophysical logs (e.g. resistivity and sonic) that are useful for a better characterization and interpretation of the breakout data.

### Earthquake focal mechanism data

This category contains crustal earthquakes with M ≥ 4, usually moment magnitude (Mw), and maximum depth of 40 km. Concerning the magnitude cut off, it is related to most of the used dataset that include focal mechanisms of M ≥ 4 earthquakes. Since even smaller events can help constraining the stress tensor^48^, in the next IPSI releases, using higher quality data, we could insert earthquakes with lower magnitude, particularly useful in areas with few data. Relatively to the depth, most of crustal earthquakes occur within the upper 20 km and we do not consider seismicity related to the still active subduction zones.

For the oldest 23 seismic events that occurred between 1908 and 1975, we consider results from the polarity solutions of earthquakes computed by a range of previous studies. For seismic events from 1976 to 2017, we account for Centroid Moment Tensor (CMT) solutions of earthquakes selected from the European-Mediterranean RCMT catalogue^49^ and the Italian CMT dataset^50,51^. For earthquakes that occurred from 2018 to present, we use the Time Domain Moment Tensor (TDMT) catalogue^52^. The focal mechanism solutions^30^ are obtained from the high-quality data of the Italian broadband and Mediterranean seismographic networks using the long-period full waveform inversion code originally proposed by ref. ^53^. Taking into account the systematic error of CMT-like solutions ( ± 14° according to ref. ^54^), range of stress orientations that would be consistent with each focal mechanism, and that the orientation of P (compression), B (null) and T (extension) axes may slightly deviate from the principal stress orientations^55^, the WSM^31,56^ assigns *C*-quality to these data (see also ref. ^25^ for a detailed explanation). Following the WSM criteria, we assign *C*-quality to all focal mechanism data.

To identify the Shmin azimuth and tectonic regime, we use the plunge of the P, T and B axes by applying the criteria of ref. ^6^, modified for Shmin (Table 2). We discard all of the focal solutions with P, T and B axes that do not define a clear tectonic regime. Although focal plane solution principal axes may not be indicative of stress axes, the possible differences between Shmin derived from P, T and B axes and Shmin from slip vectors lie within the error of the attributed quality category, as shown in ref. ^16^. Moreover, regional compilations show that the average orientations of P, B, and T axes determined from a number of earthquakes yield a good indication of the stress orientation throughout a region (e.g. ref. ^57^). Thus, the orientation of the kinematic axes (P, B, and T) is assumed to coincide with those of the dynamic axes (S1, S2 and S3).

**Table 2:** Assignment of tectonic regime and horizontal stress orientations (SHmax and Shmin) for earthquake focal mechanisms and inversions from plunges of P, B, T or S1, S2, S3 axis (according to ref. ^6^ and ref. ^15^).

P/S1 axis	B/S2 axis	T/S3 axis	Tectonic regime	SHmax	Shmin
plunge ≥ 52°		plunge ≤ 35°	NF	azimuth of B	azimuth of T
40° ≤ plunge < 52°		plunge ≤ 20°	NS	azimuth of T + 90°	azimuth of T
plunge < 40°	plunge ≥ 45°	plunge ≤ 20°	SS	azimuth of T + 90°	azimuth of T
plunge ≤ 20°	plunge ≥ 45°	plunge < 40°	SS	azimuth of P	azimuth of P + 90°
plunge ≤ 20°		40° ≤ plunge < 52°	TS	azimuth of P	azimuth of P + 90°
plunge ≤ 35°		plunge ≥ 52°	TF	azimuth of P	azimuth of B

When selecting an element on the IPSI website, a pop-up window provides the related identification code and summary information from the data record. The upper part provides the type, quality, stress orientation, tectonic regime, geographic coordinates, date of the first online publication and a reference to the focal mechanism data source. The lower part provides the earthquake date, magnitude, depth, strike, dip and rake of the two nodal planes (Fig. 3b).

### Formal inversion of earthquake focal mechanism data

This category includes stress orientations determined from the inversion of P, B, and T axes of diffuse seismicity. The data are located in close geographic proximity where a homogeneous stress field can be hypothesised and where formal inversions of more than 8 well-constrained single events are present with a standard deviation or misfit angle less than 20°. We account for literature data that satisfy the above criteria and assign them as mostly *B*-quality.

We use the same method as that for earthquake focal mechanisms to define the tectonic regime and corresponding Shmin for each inversion (Table 2). When selecting an element on the IPSI website, a pop-up window provides the related identification code and summary information from the data record. The upper part provides the type, quality, stress orientation, tectonic regime, geographic coordinates, date of the first online publication and the reference of the inversion of focal mechanisms. The lower part provides the code name, region, number of earthquakes used in the inversion procedure, year range of the events, magnitude range, depth range, misfit, azimuth and dip of S1, S2 and S3 (Fig. 3c).

### Fault slip data

This stress indicator category includes data from single faults with known attitude and primary sense of slip. As described in ref. ^16^, we do not include fault data related to earthquakes whose focal mechanisms are available, the stress information can be found in the focal mechanism category. The only exception concerns the latest surface faulting related to the 2016 central Italy seismic sequence. Starting from that date, with the aim to provide more complete information, we decided to include surface faulting data even if focal mechanism solutions are available. We are aware that this choice requires more attention when using the entire dataset to avoid duplicates, but also allows the use of complete fault category data only if needed.

Each fault, strike, dip, slip and kinematics provided in the original studies are used to define the Shmin orientation and tectonic regime in the same way as the focal mechanism data. When the slip is unknown, the Shmin orientation is assumed to be perpendicular to the fault strike for normal faults. We assign *C*-quality to all fault data, as suggested by the WSM guidelines.

When selecting an element on the IPSI website, a pop-up window provides the related identification code and summary information from the data record. The upper part provides the type, quality, stress orientation, tectonic regime, geographic coordinates, date of the first online publication and a reference to the field studies of the fault. The lower part provides the region and name (Fig. 3d).

## Data Records

The dataset can be consulted and downloaded from the INGV website (10.13127/IPSI.1.4), which is typically updated once per year or more if necessary by adding the focal mechanisms of recent earthquakes or other newly analysed data. The dataset presently contains 928 records: 423 borehole breakouts; 459 earthquake focal mechanisms; 27 focal mechanism inversions; 18 faults and 1 overcoring^58,59^ not described here because it was inherited from the WSM database^36^.

The full dataset of IPSI version 1.4 is also published within the PANGAEA® Data Publisher^60^ as five tables within XLSX files, one for each stress indicator group and a reference/description file. These files include:

- Borehole breakout data;
- Earthquake focal mechanism data;
- Formal inversion of earthquake focal mechanism data;
- Fault slip data;
- Overcoring data.

Each table contains the following fields, as described below (Table 3).

**Table 3:** Examples of data records of borehole breakout (BO) and earthquake focal mechanism (FMS).

Field	BO field name	BO record	FMS field name	FMS record
1	Id	BO366	Id	FMS440
2	N	366	N	0440
3	Type	BO	Type	FMS
4	Lat	38,94	Lat	42,09
5	Lon	17,28	Lon	13,32
6	Sh	161	Sh	38
7	SH	251	SH	306
8	Q	B	Q	C
9	TR	U	TR	NF
10	Reference1_original	Montone & Mariucci 2016	Reference1_original	RCMT Catalog
11	Reference2_last	Montone & Mariucci 2016	Reference2_last	IPSI 1.3 2019
12	WEB_date	01/07/2017	WEB_date	15/01/2018
13	Update	05/07/2018	Update	15/07/2019
14	sd	14	Download_date_from_catalog	01/07/2019
15	BO_top	1507	Date_eq	10/09/2017 T19:58
16	BO_bottom	1977	Mw	4,1
17	available		Depth_(km)	8,0
18	UNMIG_well_code	3364	strike1	139
19	Year	2000	dip1	32
20	Depth	2426	rake1	-75
21	Well_name	Lulu 1	strike2	301
22			dip2	59
23			rake2	−100

### Fields common to all data

1. **Id**: Identification code of the data record. Letters indicate the indicator type (field 3) and ordinal number (field 2).
2. **N**: Ordinal number of the data record.
3. **Type**: Stress indicator type following the WSM classification^31^: BO, borehole breakout; FMS, single focal mechanism; FMF, formal inversion of focal mechanisms; GFS, faults; OC, overcoring.
4. **Lat**: Latitude north in decimal degrees within the WGS_1984 geographic coordinate system rounded to two decimal places.
5. **Lon**: Longitude east in decimal degrees within the WGS_1984 geographic coordinate system rounded to two decimal places.
6. **Sh**: Computed minimum horizontal stress orientation.
7. **SH**: Computed maximum horizontal stress orientation.
8. **Q**: Assigned stress orientation quality from *A* (best) to *E* according to the WSM classification^31^.
9. **TR**: Defined tectonic regime. Two letter code: NF, normal fault; SS, strike-slip fault; TF, thrust fault; TS, thrust-strike fault; NS, normal-strike fault; U, unknown.
10. **Reference1_original**: Source of the raw data (e.g. earthquake focal mechanism catalogues) or the first paper containing the data record (for breakout data).
11. **Reference2_last**: Reference of the last update of the whole dataset or last reference for the data record.
12. **WEB_date**: Date of the first online inclusion in the IPSI database (dd/mm/yyyy).
13. **Update**: Most recent update of the data record, if necessary (dd/mm/yyyy).

### Fields for borehole breakout data (BO) only

14. **sd**: Standard deviation of horizontal stress orientation.
15. **BO_top**: Shallowest breakout depth (m). Measured depth from rotary table.
16. **BO_bottom**: Deepest breakout depth (m). Measured depth from rotary table.
17. **available**: Availability of the well log at the Italian Ministry of Economic Development (‘no’ or empty field).
18. **UNMIG_well_code**: Well Code number from the National Mining Office for hydrocarbon and geothermal energy of the Italian Ministry of Economic Development.
19. **Year**: Year of drilling (for available wells only).
20. **Depth**: Total vertical depth (m) from rotary table (for available wells only).
21. **Well_Name**: Well name (for available wells only).

### Fields for earthquake focal mechanism data (FMS) only

14. **Download_date_from_catalog**: Date of download from the focal mechanism catalogue (dd/mm/yyyy).
15. **Date_eq**: Earthquake date (dd/mm/yyyy) and time (hh:mm).
16. **Mw**: Earthquake magnitude to one decimal.
17. **Depth_(km)**: Earthquake hypocentral depth (km) to one decimal. Depth below sea level.
18. **strike1**: Strike of nodal plane 1, integer.
19. **dip1**: Dip of nodal plane 1, integer.
20. **rake1**: Rake of nodal plane 1, integer.
21. **strike2**: Strike of nodal plane 2, integer.
22. **dip2**: Dip of nodal plane 2, integer.
23. **rake2**: Rake of nodal plane 2, integer.

### Fields for formal inversion data (FMF) only

14. **Name**: Code identifying the inversion, combination of letters and numbers according to the data source or given by database authors.
15. **Region**: Italian region or zone where the data are located.
16. **Events_num**: Number of events used for the inversion.
17. **Year**: Year or range of years of the earthquakes used for the inversion.
18. **M**: Range of magnitude of the events used for the inversion.
19. **Depth_(km)**: Range of depth (km) of the events used for the inversion. Depth below sea level.
20. **Misfit**: Value indicating the reliability of the inversion (if available).
21. **σ1_(az/dip)**: Azimuth/dip of the major stress axis.
22. **σ2_(az/dip)**: Azimuth/dip of the intermediate stress axis.
23. **σ3_(az/dip)**: Azimuth/dip of the minor stress axis.

### Fields for fault data (GFS) only

14. **Region**: Italian region where the fault is located.
15. **Fault_Name**: Fault name assigned by the authors in field 10 (“Reference1_original”).

### Field for overcoring data (OC) only

14. **Locality**: Zone where the data are located.

## Technical Validation

Each stress category included in the IPSI database follows rigorous recognised and shared technical procedures. Concerning the borehole breakouts, we apply standard operating procedures for the data analysis. For example, we compare the results of the analysis performed on the same borehole by different analysts or by using different kinds of data (e.g. digital vs. paper logs). In both cases, the results are consistent within the error of each quality category. With regard to fault data, the collected field measurements are affected by uncertainties owing to qualitative and quantitative factors, such as environmental conditions, representativeness of the measured object and instrument maintenance, precision and accuracy^61^. With regard to the earthquake data, we refer to different worldwide catalogues (Italian CMT dataset, European Mediterranean RCMT catalogue, quick regional moment tensors and the TDMT-INGV catalogue) and to compare the results of the analysis before their inclusion in the database.

Notwithstanding the standard procedures and measurement accuracy, different uncertainties can affect the data. To account for this, we use the stress indicator quality ranking system provided by WSM^31^ that is accepted and tested worldwide to ensure reliability and global comparability with providing error estimates to each datum. As mentioned, a quality category from *A* to *E* (best to worst) is assigned to each stress data orientation record mainly on the basis on the number of measurements, accuracy, and depth. For these reasons, only *A*-, *B*- and *C*-quality stress indicators are usually considered reliable in the analysis of stress patterns and interpretation of crustal geodynamic processes.

## Usage Notes

The contents of the database are easily accessible through a user-friendly web interface. From the upper bar, the user can open pop-up windows or files with a wide range of general information. The legend on the left side allows users to obtain information on the dataset and plot on the map all data or select categories of stress indicators and data quality. The entire IPSI dataset can be downloaded in different formats from the lower-left window including CSV (comma separated values), XLS (MS Excel), KML (Google Earth, Keyhole markup language), MIF (Map Info), SHP (ESRI) and TXT. The latter contains only latitude, longitude and SHmax from *A*–*C* data and is ready for use within SHINE software (http://shine.rm.ingv.it/) to perform simple data interpolations.

## Data Availability

The code used in the IPSI 1.4 web interface is open and based on HTML language. The server procedures are developed in PHP open-source language and the client-side procedures are developed in JavaScript language with specific features of jQuery (http://jquery.com/) and Google Maps API (https://developers.google.com/maps/).
